# Ensemble Methods for MiRNA Target Prediction from Expression Data

**DOI:** 10.1371/journal.pone.0131627

**Published:** 2015-06-26

**Authors:** Thuc Duy Le, Junpeng Zhang, Lin Liu, Jiuyong Li

**Affiliations:** 1 School of Information Technology and Mathematical Sciences, University of South Australia, Adelaide, South Australia, Australia; 2 School of Engineering, Dali University, Dali, China; IPMC, CNRS UMR 7275 UNS, FRANCE

## Abstract

**Background:**

microRNAs (miRNAs) are short regulatory RNAs that are involved in several diseases, including cancers. Identifying miRNA functions is very important in understanding disease mechanisms and determining the efficacy of drugs. An increasing number of computational methods have been developed to explore miRNA functions by inferring the miRNA-mRNA regulatory relationships from data. Each of the methods is developed based on some assumptions and constraints, for instance, assuming linear relationships between variables. For such reasons, computational methods are often subject to the problem of inconsistent performance across different datasets. On the other hand, ensemble methods integrate the results from individual methods and have been proved to outperform each of their individual component methods in theory.

**Results:**

In this paper, we investigate the performance of some ensemble methods over the commonly used miRNA target prediction methods. We apply eight different popular miRNA target prediction methods to three cancer datasets, and compare their performance with the ensemble methods which integrate the results from each combination of the individual methods. The validation results using experimentally confirmed databases show that the results of the ensemble methods complement those obtained by the individual methods and the ensemble methods perform better than the individual methods across different datasets. The ensemble method, Pearson+IDA+Lasso, which combines methods in different approaches, including a correlation method, a causal inference method, and a regression method, is the best performed ensemble method in this study. Further analysis of the results of this ensemble method shows that the ensemble method can obtain more targets which could not be found by any of the single methods, and the discovered targets are more statistically significant and functionally enriched. The source codes, datasets, miRNA target predictions by all methods, and the ground truth for validation are available in the Supplementary materials.

## Introduction

miRNAs are important endogenous non-coding RNAs that regulate gene expression by promoting mRNA degradation and repressing translation [1]. They regulate target mRNAs post-transcriptionally by base pairing to complementary sequences in the 3’-untranslated region (3’UTR) of the mRNAs [1–4]. miRNAs have also been observed to target genes through sites in the 5’UTR and sometimes in the open reading frames (ORFs) [5]. They are involved in several cancer-related processes such as proliferation [6, 7], metabolism [8], differentiation [9], development [10], apoptosis [11], cellular signaling [12], and even cancer development and progression [1, 13]. Identifying miRNA functions helps elucidate the complex mechanisms of fatal diseases and assists with the design of drugs for treatments.

Several methods have been proposed to identify miRNA targets using sequence data [14–16], which are based on sequence complementarity and/or structural stability of the putative duplex. Although these methods can produce a list of potential target genes, the results may contain a high-rate of false positives and false negatives [17]. Moreover, the results predicted using different methods are often inconsistent [17]. A major reason for this is that the miRNA binding sites are small, thus they do not support statistically significant predictions, and a small difference in the algorithms can lead to a great diversity in the results [17, 18]. In addition, the predictions are static, and the predicted targets of a certain miRNA might not be expressed at all in a specific condition [19].

In recent years, various computational methods have been devised to incorporate expression profiles into the study of miRNA-mRNA regulatory relationships. The principle of these methods is that if a gene is regulated by a miRNA, a correlation should show between the expression levels of the gene and the miRNA. The first stream of the research saw various methods developed to identify co-expressed miRNAs and mRNAs. Some highlights of those methods are correlation analysis [20, 21], regression models [22, 23], population-based probabilistic learning model [24], and rule based methods [25]. In the second stream, attempts were made to predict the regulatory networks of miRNAs and mRNAs for specific biological processes, using methods such as Bayesian network learning [26–28] and causal inference techniques [29–31].

It has been shown that computational methods may perform inconsistently in inferring gene regulatory networks with different datasets [32]. Each method usually has a set of assumptions on data when building the model. These assumptions may be suitable for some datasets but may be violated in the others. For example, correlation based methods assume linear relationships between variables. In reality, however, many relationships are not linear, or even cannot be approximated using linear relationships. Therefore, these methods may not perform well when the underlying relationships are not linear. As shown in the **Results** section, our experiments on different matched miRNA and mRNA expression datasets confirm the inconsistent performance of the target prediction methods. Therefore, it is important to design more reliable methods for miRNA target prediction.

On the other hand, ensemble methods which integrate different methods have been shown to perform better than all the individual component methods [32]. Different miRNA target prediction methods have their own pros and cons, and their results may complement each other. Therefore, combining the predictions of multiple methods would improve the prediction power.

In this paper, we firstly compare the performance of eight popular miRNA target prediction methods with three different cancer gene expression datasets. We then construct the ensemble methods such that each of the ensembles integrates a different combination of the top five individual methods, and investigate their performance against the top five individual performers. The experimental results show that the top performed ensemble methods are better than all individual methods. Especially, the method that combines a correlation method (Pearson), a causal inference method (IDA), and a regression method (Lasso) is the best method in the study. Importantly, our experimental results show that different individual methods infer complementary results, suggesting their own merits, and the top ensemble methods outperform the individual methods by including those complementary results. Although there is no complete ground truth of miRNA target prediction, the results show that ensemble methods can find some confirmed interactions in the incomplete ground truth that existing individual methods fail to discover. Therefore, using ensemble methods, we would receive more reliable results than existing individual methods.

Related to our work, there have been several methods [33–35] of integrating the results of multiple sequence-based prediction methods. For examples, Zhang and Verbeek [33] presented a comparison study of integrating three sequence-based prediction methods, miRanda [14], TargetScanS [15], and RNAhybrid [36], using different integrating approaches, including the “low level” approaches which deal with raw data features directly and the “high level” approaches which combine predictions from different methods. The “high level” approaches in this study, e.g. cross entropy aggregation [37], share the same purpose with our study and can be adapted for integrating predicted results from expression-based methods. Our experiment results, however, show that the ensemble methods that are based on Borda count election are better than the cross entropy approach for the datasets in this study. Shirdel et al. [35] collated commonly used sequence-based prediction databases and created a database called mirDIP (http://ophid.utoronto.ca/mirDIP/). The database allows users to explore miRNA targets using one or the combination of multiple prediction databases. They also used mirDIP to explore miRNA-target interactions and the signaling pathway networks. This is a good resource for miRNA research, but the aim is different from that of our study as we explore the prediction power of the methods using gene expression data. Moreover, our approach not only seeks to outperform the individual methods, but also aims to discover the interactions that individual methods may fail to identify. In another direction, Pio et al. [34] proposed a semi-supervised method that was based on support vector machine to predict the miRNA targets. They used an experimentally confirmed database, miRTarBase [38], together with the results of ten sequence-based prediction methods in mirDIP as the training data, and used another experimentally confirmed database, Tarbase [39], as the ground truth to evaluate the model. This is different from our approach since we integrate unsupervised methods to form a more accurate model. In our work, we utilise both miRTarBase and Tarbase for validation due to the sparseness in the number of confirmed interactions, and expression data are used for training the individual methods.

The datasets we work on are the matched miRNA and mRNA expression profiles in the NCI-60 cancer cell lines datasets (EMT), the multi-class cancer (MCC) datasets, and the 51 human breast cancer cell lines datasets (BR51). We use the experimentally confirmed miRNA target databases to validate the effectiveness of the methods. The results indicate that integrating different miRNA target prediction methods would generate reliable prediction results. We report miRNA target predictions and validated targets for all the individual and the ensemble methods discussed in the paper. We provide the R scripts for constructing ensemble methods from individual methods which can be used to integrate other miRNA target prediction methods.

## Materials and Methods

### Datasets

#### NCI-60 data for Epithelial to Mesenchymal Transition (EMT)

EMT is part of the process of tissue remodelling during embryonic development and wound healing [40], and during cancer progression [41]. The NCI-60 data were profiled from the 60 cancer cell lines of the drug screening panel of human cancer cell lines at the National Cancer Institute. The miRNA expression profiles are from Søkilde et al. [42]. They are available at http://www.ncbi.nlm.nih.gov/geo/query/acc.cgi?acc=GSE26375. The mRNA expression profiles for NCI-60 were obtained from ArrayExpress http://www.ebi.ac.uk/arrayexpress, accession number E-GEOD-5720. We use the work in [43] to categorise the samples into epithelial (11 samples) and mesenchymal (36 samples).

We perform differential gene expression analysis on the gene expression profiles to identify differentially expressed miRNAs and mRNAs between the epithelial and mesenchymal samples. In this work, the *limma* package [44] of Bioconductor is used for the analysis. As a result of the analysis, 35 probes of miRNAs and 1154 probes of mRNAs are identified to be differentially expressed at significant level (adjusted *p*-value < 0.05, adjusted by Benjamini–Hochberg (BH) method). The detailed result can be found in S1 File.

#### Multi-class cancer (MCC) data

The MCC dataset includes normal and cancerous samples from bladder, breast, colon, kidney, lung, pancreas, prostate and uterus. As for the EMT data, we use the differential gene expression analysis to find the differentially expressed miRNAs and mRNAs between normal and cancerous samples. The miRNA expression profiles are from [45], and 108 probes are found to display differential expression at significant level (*p*-value < 0.05, adjusted by BH method). The mRNA data are from [46], with 1860 mRNA probes being identified to display significant differential expression. The detailed result can be found in S1 File.

#### Data of the 51 human breast cancer cell lines (BR51)

Breast cancer is a complex disease which involves several different sub-types. In this work, we use the BR51 data from [47]. The miRNA data were downloaded from [47] and the mRNA gene expression data are available at: http://www.ncbi.nlm.nih.govgeoqueryacc.cgiacc=GSE41313.

We use the samples categorised results from [47] to divide the samples into luminal and basal groups. There are 27 samples in the luminal group and 23 samples in the basal group. In these datasets, the changes in expression levels of the mRNAs in the two groups are very significant, while the changes in the case of miRNAs are small. In total, 92 miRNAs (adjusted *p*-value < 0.2) and 1500 mRNAs (adjusted *p*-value < 0.0001) are identified to be differentially expressed. To cover the important miRNAs mentioned in the analysis of [47] and to have a manageable number of mRNAs for the computational methods, we choose different thresholds of adjusted *p*-values in the differential gene expression analysis. Details of the datasets are available in S1 File.

### Methods of miRNA target prediction

In this section, we introduce the commonly used methods for miRNA target prediction using gene expression data.

Pearson’s correlation coefficient is the commonly used measure for the strength of the association between a pair of variables. For the miRNA target prediction problem, the correlations in the expression levels between pairs of miRNA and mRNA are calculated. The miRNA-mRNA pairs are ranked based on the correlation coefficient values. As miRNAs mainly down-regulate mRNAs, the negative correlations are ranked at the top (according to their absolute values).

However, Pearson correlation is designed to capture only linear associations, and its usefulness is greatly reduced when the associations are non-linear [48]. To deal with this problem, the maximal information coefficient (MIC) [49] has been proposed to measure a wide range of associations, including linear and non-linear relationships, with comparable scores. Therefore, both Pearson and MIC are selected in our comparison study.

Meanwhile, Lasso and Elastic-net are high-dimensional regression techniques which can be used to infer the associations between variables. In our problem, the expression level of each mRNA is presented as a linear expression of the expression levels of all miRNAs, and the coefficient of a miRNA in the regression model is used as the association strength between the miRNA and the mRNA. For each mRNA, we can perform Lasso and Elastic-net regression on all the miRNAs, using the R package *glmnet* [50]. Similar to the Pearson correlation coefficient method, the miRNA-mRNA pairs that have negative effects are ranked at the top of the ranking list to favour the down regulation.

In another direction, Maathuis et al. [51, 52] proposed a causal inference method, called IDA, which estimates the causal effect that a variable has on the other. The estimated causal effects simulate the effects of randomised controlled experiments. Recently, Le et al. [29] applied this method to gene expression datasets to infer the causal (regulatory) relationships between miRNAs and mRNAs. The discovered miRNA-mRNA causal regulatory relationships were found to have a large portion of overlap with the results of the follow-up gene knockdown experiments. Therefore, we include this method in our study.

Z-score [53] is a network inference method to estimate the effects of gene knockout experiment. When we knockout a miRNA, Z-score describes the normalised deviation of the expression level of each gene from the average expression of all genes. However, in our study, we use observational data, i.e. gene expression data, as the input dataset. Therefore, the Z-score method is modified where we assume that the minimum expression value of the miRNA in a profile indicates the knockout experiment. In other words, we firstly locate the sample that contain the minimum value of the miRNA, then we use the expression value of the mRNA in the sample to calculate Z-score.

ProMISe [54] assumes that there is a competition between miRNAs to be attracted by a mRNA and between mRNAs to attract a miRNA for interactions. The method estimates the probability of a mRNA to be a target of a miRNA by taking the competition between mRNAs and between miRNAs into account. The tool was downloaded from Bioconductor at: www.bioconductor.org/packages/release/bioc/html/Roleswitch.html.

GenMiR++ [26] is a well-cited and commonly used method for miRNA target prediction. GenMiR++ is a Bayesian network parameter learning method to infer the probabilities of targets of being true by approximating the posterior with variational inference techniques. In the model, the changes in mRNA expression level are encoded by the summation of negatively weighted changes of the expression level of their regulators miRNAs. The Matlab code of GenMiR++ was obtained from http://www.psi.toronto.edu/genmir/.

### Ensemble methods for miRNA target prediction

Ensemble methods are designed to take the advantages of the individual methods, and to compensate for their drawbacks. Specifically, individual methods rank the miRNA-mRNA interactions based on some criteria, e.g the strength of the correlation coefficients, and ensemble methods integrate the rankings from different methods to form a new ranking. There are different strategies for integrating different rankings, such as taking the intersection, the union, or the average of the top *k* results predicted by individual methods. Marbach et al. [32] have proved that the Borda count election method is a simple yet effective method for integrating the results from different individual methods. Borda count election is presented by Jean-Charles de Borda as a method to select candidates in a democratic election by choosing the candidate with the best average rank. In this paper, we apply the Borda count election method for forming ensemble methods. Please note that using Borda average rank of multiple methods does not result in a method which averages the performance of the individual methods. In fact, the aim is to design a method that outperforms the individual methods, and the method is inspired by the following theorem which was proved in [32].

**Theorem 1** [32]: *Let M_i_, i* = 1…*n, be the prediction methods. If each M_i_*, 1 ≤ *i* ≤ *n, has better than random predictive power, then the community (ensemble) method of applying Borda count election method can outperform the best individual method.*

We summarise the procedure of the ensemble methods in the following, and the *R* source code for the ensemble methods which use Borda count election is available in S2 File.

**Step 1**: With a dataset, identify differentially expressed miRNAs and mRNAs between different biological conditions (this step is common for all methods in the comparison).

**Step 2**: For each differentially expressed miRNA, we use each of the individual methods (Pearson, IDA, MIC, Lasso, Elastic, Z-score, ProMISe, and GenMiR++) respectively to produce the rankings of the differentially expressed mRNAs (as the predicted targets of the miRNA). Then, we select top five performers in identifying miRNA targets based on the validation results using experimentally confirmed targets.

**Step 3**: Apply Borda rank election to the rankings from Step 2 for each miRNA to produce a single ranking list of elected mRNAs with respect to the miRNA. We then extract the top-ranked genes from the list as the final output, i.e. the potential target genes for the given miRNA.

We have implemented the Borda count election method for integrating all combinations of the selected top individual miRNA target prediction methods. In our implementation, we do not use the whole ranking list produced by each method to produce the ranking for the ensemble methods, but only use the top *k* (users defined) predicted targets of a miRNA produced by individual methods. The ensemble methods are designed to appreciate the genes that are predicted within the top *k* predicted targets across each individual component method. Genes that are not in the top *k* predicted targets of a method is assigned the maximal ranking for that method, i.e. the maximum number of genes in the dataset.

### Ground truth for validation

It is difficult to validate computation results, as the number of experimentally confirmed targets of miRNAs are still limited and there is no complete ground-truth for evaluating and comparing different computational methods [55]. In this paper, we use the union of four popular databases, Tarbase v6.0 [39], miRecords v2013 [56], miRWalk v2.0 [57], and miRTarBase v4.5 [38] to validate the predictions of the methods for all miRNAs in the EMT, MCC and BR51 datasets. Tarbase, miRecords, and miRTarBase include verified interactions that are manually curated from the literature, and miRWalk contains both the predicted and the experimentally validated interactions. Respectively for Tarbase, miRecords, miRWalk, and miRTarBase, we have 20095 interactions with 228 miRNAs, 21590 interactions with 195 miRNAs, 1710 interactions with 226 miRNAs, and 37372 interactions with 576 miRNAs. Table 1 shows the number of overlapping interactions between each pair of the databases. After removing the duplicates, we have in total 62858 unique interactions to be used in the validation (details in S3 File). We refer to those interactions as “confirmed interactions” in this paper.

**Table 1:** Number of overlapping interactions between databases.

	Tarbase	miRecords	miRWalk	miRTarBase
Tarbase	20095	593	646	15100
miRecords		1710	537	1147
miRWalk			21590	1482
miRTarBase				37372

## Results

### Individual methods perform inconsistently across datasets

**Fig 1:**
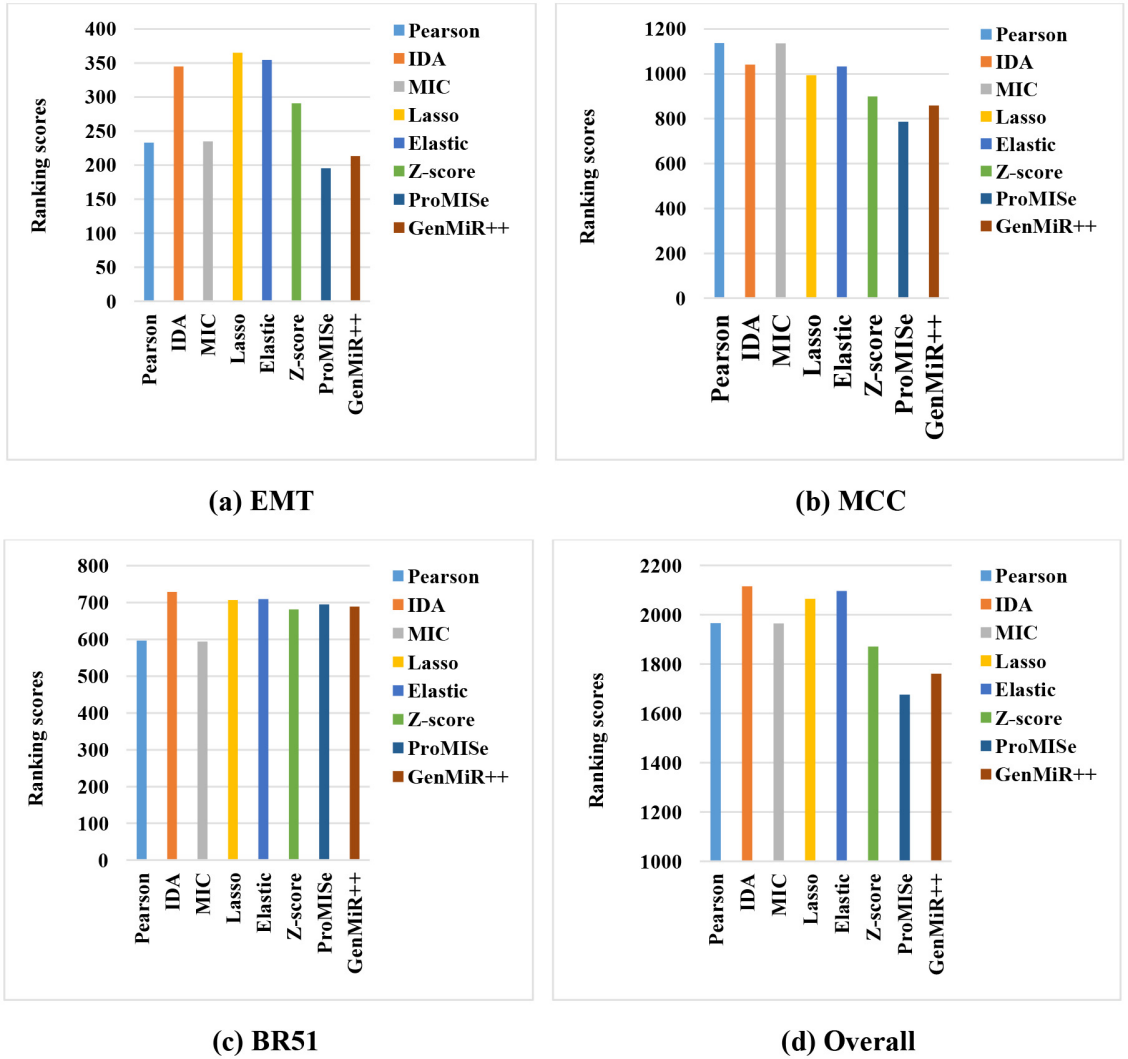
Comparison of eight individual miRNA target prediction methods.

In this section, we compare the performance of eight individual methods on the three datasets. For each miRNA, we extract the top 100, 200, 300, and 400 target genes ranked by each method for validation. We only keep in the validations the miRNAs that have at least one confirmed target predicted by all the methods. We then compare the performance of the methods for each miRNA based on the number of confirmed targets using defined ground truth. As we have eight methods, with respect to each miRNA, we score each method using a number (called ranking score) in the range of 1 to 8, with 8 indicating the best method and 1 the worst method. Finally, we calculate the ranking score of each method for a dataset by summing up its scores for all miRNAs. The higher the ranking score of a method, the better the method is. Fig 1 shows the comparison in terms of their ranking scores in each of the datasets (Fig 1(a), 1(b) and 1(c)), and the overall ranking score in all three datasets (Fig 1(d)). The overall ranking score is calculated by summing up the scores in all miRNAs in all three datasets.

From the figure, we can see that there is no superior method outperforming the other methods in all datasets. Pearson correlation and MIC perform very well in the MCC dataset, but their performance is not very good in the other two datasets. Meanwhile, IDA, Lasso, and Elastic-net perform consistently in the three datasets, and therefore they are the best performers overall as shown in Fig 1(d). ProMISe and GenMiR++ perform similarly to the top performers (IDA, Lasso, and Elastic-net) in the BR51 dataset, but they are the bottom two methods in EMT and MCC. The possible reason for GenemiR++ and ProMISe having performed badly is that they are originally designed to also use the target information predicted based on sequence data. When they are applied to gene expression data alone, their performance is not as good as the other methods.

Overall, IDA is the best method and ProMISe is the worst method in terms of finding confirmed miRNA targets from expression data. However, note that each method has its own merits as they may discover different sets of miRNA targets [55]. In the following section, we investigate the performance of the ensemble methods with the hypothesis that they may outperform all the individual methods.

### Top ensemble methods are better than individual methods

In this section, we investigate the performance of the ensemble methods constructed based on different combinations of the individual miRNA target prediction methods. As our aim is to utilise the strength of each individual method across different datasets, we remove ProMISe and GenMiR++ from the candidates of forming ensemble methods, due to their poor performance in all the gene expression datasets. Therefore, we do not discuss the results of the ensemble methods that include ProMiSe and GenMiR++ in this study. However, we report the comparison results of the ensemble method that includes ProMise, GenMiR++ and other methods in S4 File. We have also observed that Lasso and Elastic-net perform similarly to each other, therefore we remove Elastic-net from the combinations for forming the ensemble methods. In the end, we have five methods (Pearson, MIC, IDA, Lasso, and Z-score) for forming ensemble methods.

We firstly combine *k* individual methods (*k* = 2, 3, 4, 5) using the Borda count election method (described in Materials and Methods) to create a level *k* ensemble method. We then select the three best performed level *k* methods (except one method for level five) and compare their performance across the three datasets. Table 2 shows the performance of the ensemble methods in terms of their ranking scores. We can see that all level 3 ensemble methods are better than any individual methods and the ensemble methods at other levels. Particularly, Pearson+IDA+Lasso is the best method in this comparison study.

**Table 2:** Rankings of top ensemble methods and individual methods.

Rank	Ranking scores	Level	Method
1	3783.5	3	Pearson+IDA+Lasso
2	3667	3	Pearson+IDA+Z-score
3	3636	3	IDA+MIC+Lasso
4	3578	4	Pearson+IDA+MIC+Lasso
5	3530	1	IDA
6	3497.5	2	IDA+Lasso
7	3489.5	4	IDA+MIC+Lasso+Z-score
8	3484.5	2	IDA+MIC
9	3459	2	Pearson+IDA
10	3432	4	Pearson+IDA+Lasso+Z-score
11	3341	1	Lasso
12	3289	5	Pearson+IDA+MIC+Lasso+Z-score
13	3218.5	1	Pearson
14	3165.5	1	MIC
15	3029	1	Z-score

We conduct the Wilcoxon signed-rank test based on the ranking scores to compare the performance of Pearson+IDA+Lasso and each of the individual methods (Pearson, IDA, Lasso). The results show that the ensemble method is significantly better than the individual methods (the p-values are 0.008, 0.026, and 0.002 for Pearson, IDA, and Lasso, respectively), showing that an ensemble method of different approaches (correlation, causality and regression in this case) results in the better-than-individual method.

The ensemble method that combines all individual methods (i.e. the level 5 ensemble method) performs better than three individual methods (Pearson, MIC and Z-score), but not better than IDA, Lasso. However, this result does not violate the conclusion in Theorem 1, which stated that the ensemble methods outperform the individual methods. The reason is that we are using an incomplete ground truth for validating the methods, and the rankings in Table 2 indicate the performance of the methods only based on the current knowledge about miRNA-mRNA interactions. However, as we will see in the next section, an ensemble method is able to obtain complementary results, i.e. an ensemble method can identify the targets that are discovered by one individual method only but not by the other individual methods. On another note, our experimental results show that the method combining all individual methods may not be better than other ensemble methods. This observation is consistent with the results in [32]. In the following section, we will show that the ensemble method that combines methods from different approaches performs better than other ensemble methods.

**Fig 2:**
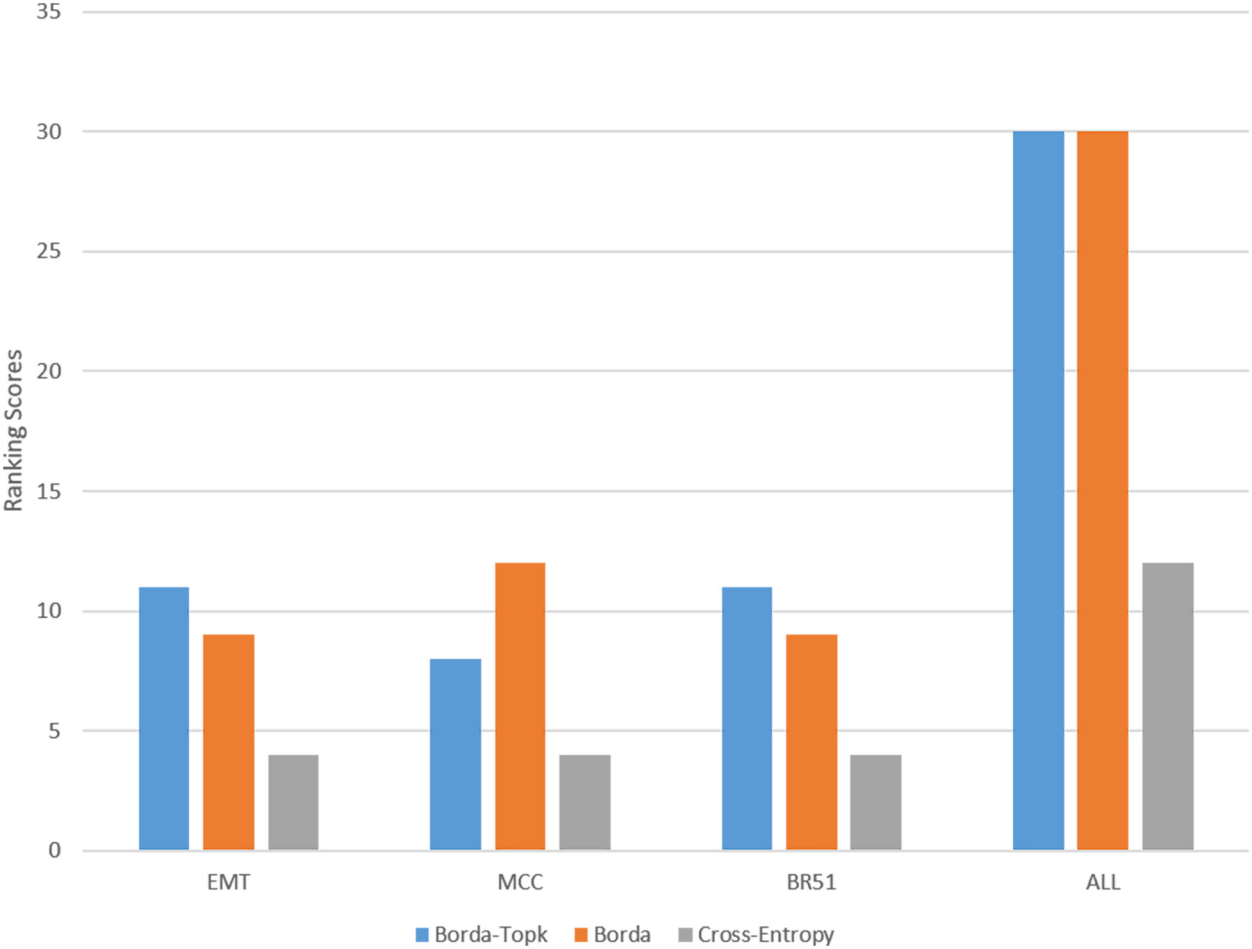
Pearson+IDA+Lasso with different integration approaches. Borda-Topk and Borda (Original Borda from [32]) are better than cross-entropy Monte Carlo in all three datasets.

The Borda count applied for top predicted targets (Borda-Topk) presented in this paper has the same principle as the Original Borda count method [32]. However, Borda-Topk provides a way for flexibility in the cut-off targets, i.e. users may only be interested in the top 200 targets of a particular miRNA. If *k* is set to the number of mRNAs in the dataset, the two methods are identical. Fig 2 shows the comparison results of the best ensemble method (Pearson+IDA+Lasso) using three different ranking integration approaches, including Borda-Topk, Original Borda, and the popular rank aggregation approach using cross-entropy Monte Carlo algorithm [37] in the *R* package called *RankAggreg* [58]. In Fig 2, we compare the ranking scores of the three approaches in each dataset and in all datasets. The ranking scores are calculated based on the number of confirmed interactions in the top 100, 200, 300 and 400 predicted targets of all miRNAs in each dataset. We can see from Fig 2 that, the Borda-Topk is the best approach in the EMT and BR51 datasets, but overall Borda-Topk and Original Borda are similar. Meanwhile, Cross-Entropy Monte Carlo is the worst approach in all three datasets. In the following sections, we discuss the results of the Borda-Topk approach.

### The ensemble methods find complementary results

**Fig 3:**
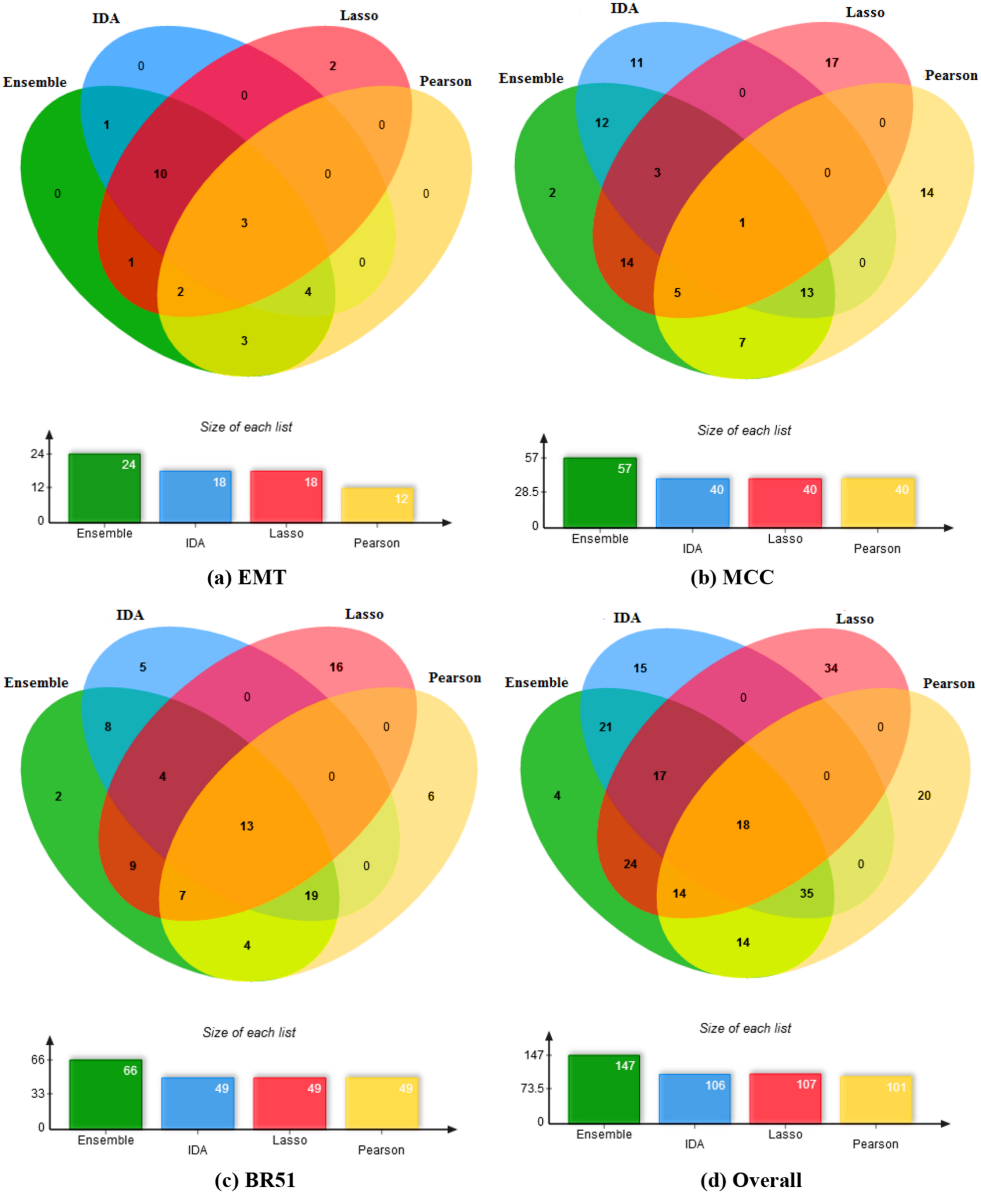
Venn diagram of the number of confirmed miRNA-mRNA interactions for the best ensemble method (Pearson+IDA+Lasso) and each individual method (Pearson, IDA and Lasso). For each miRNA, we extract top 200 target genes ranked by each method for validation.

We hypothesise that the ensemble methods are better than individual methods, because the ensemble methods can produce results which are complementary to the results of different individual methods. To have a closer look at this hypothesis, we extract the results predicted by the best ensemble method (Pearson+IDA+Lasso) and compare those results with the predictions by each individual method (Pearson, IDA and Lasso). The complementary characteristic is shown in most of the miRNA cases, especially there are many cases in which the results of the ensemble method include confirmed interactions that are not all discovered by a single individual method and therefore the ensemble method performs better than the individual methods. Such cases are shown in 4 miRNAs in EMT (miR-1180, miR-141, miR-18a and miR-96), 7 miRNAs in MCC (miR-197, miR-19a, miR-23a, miR-30a, miR-32, miR-98 and miR-9), and 6 miRNAs in BR51 (miR-125b, miR-196a, miR-21*, miR-27a, miR-30a and miR-342-5p). Fig 3 shows the comparison of the methods in terms of the number of confirmed miRNA-mRNA interactions in the top 200 targets of the miRNAs in each dataset (Fig 3(a), 3(b) and 3(c)), and the overall number in all three datasets (Fig 3(d)).

From Fig 3, the ensemble method has the largest number of confirmed miRNA-mRNA interactions in each dataset, and include most of the confirmed interactions that an individual method can discover. The advantage of the ensemble method would enhance the results of miRNA target predictions. The details of confirmed miRNA-mRNA interactions identified by each method in the three datasets can be found in S4 File.

### Targets identified by Pearson+IDA+Lasso are statistically and functionally enriched

In this section, we firstly conduct the statistical analysis of the predictions by Pearson+IDA+Lasso based on *z*-score (standard score). The *z*-score reflects the performance of a prediction method in finding confirmed targets compared to the expected rate in the ground truth, it is defined as follows:

$$z-\text{score}=\frac{x-\mu }{\sigma /\sqrt{n}},$$

where *x* is the probability of finding confirmed interactions by a method, i.e. the ratio of number of confirmed interactions and number of all possible miRNA-mRNA interactions in a dataset (*n*); *μ* is the expected probability of confirmed interactions in the ground truth, i.e. the ratio of confirmed interactions in the ground truth and all possible miRNA-mRNA interactions in the ground truth; and *σ* is the standard deviation of the population (the ground truth), and is calculated assuming Bernoulli distribution:

$$\sigma =\sqrt{\mu (1-\mu )}$$

**Fig 4:**
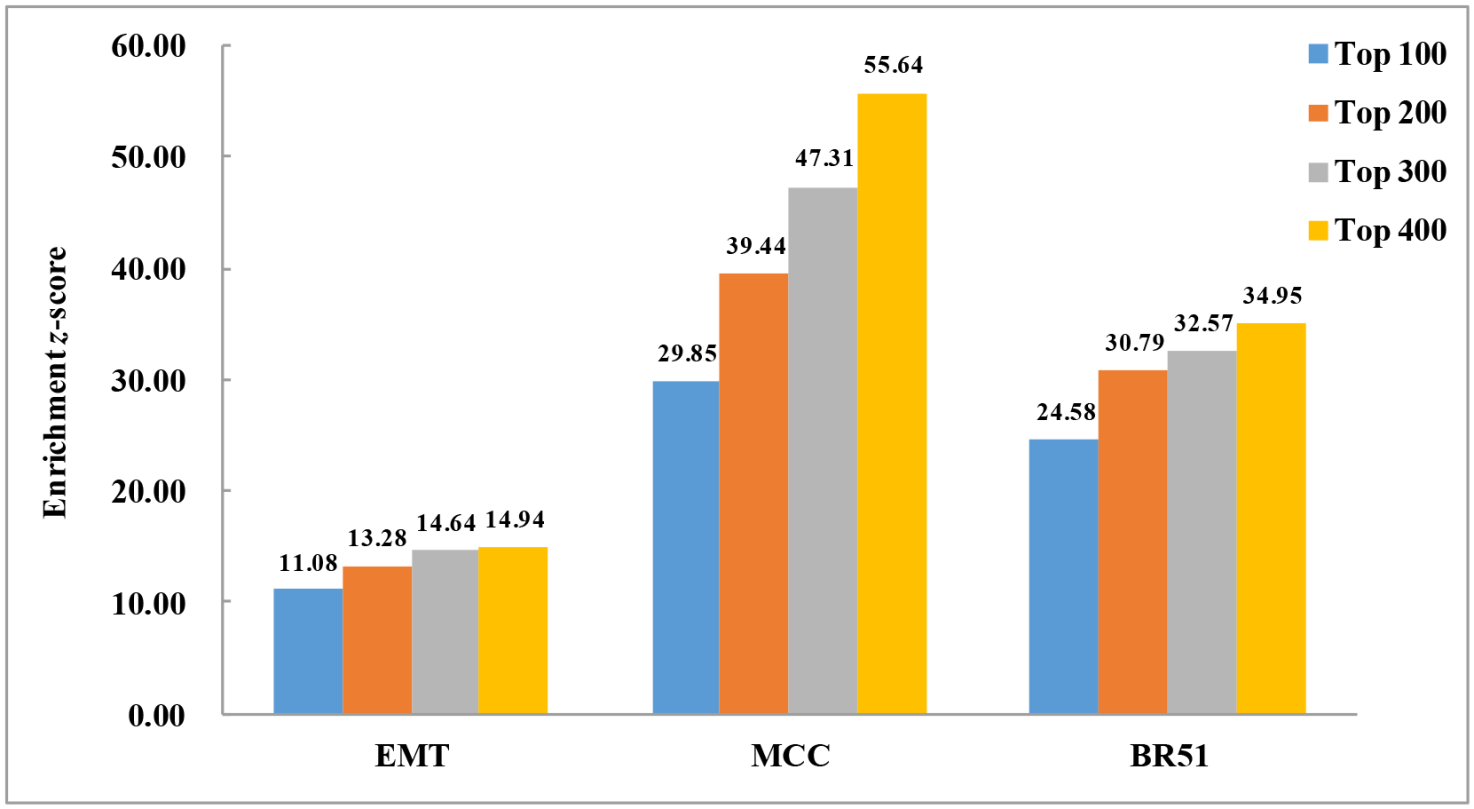
Statistical enrichment analysis of the best ensemble method (Pearson+IDA+Lasso) in EMT, MCC and BR51 datsets. For each miRNA, we extract top 100, 200, 300 and 400 target genes for the analysis.

Based on the *z*-score formula, the higher the *z*-score is, the more significant the prediction results are. As illustrated in Fig 4, the enrichment results of the ensemble method in the three datasets are high (*z*-score>10), suggesting that the results of the ensemble method are not random and they are significant, and the method is effective in recovering confirmed interactions.

We also use the GeneCodis [59] (the online tool at http://genecodis.cnb.csic.es/analysis) to conduct pathway enrichment analysis of target genes with the focus on significant KEGG (Kyoto Encyclopedia of Genes and Genomes) [60] pathways (adjusted *p*-value<0.05). We select the genes in the top 1000 miRNA-mRNA interactions identified by the method in each dataset for the analysis. As shown in Table 3, the top 10 enrichment KEGG pathways in the three datasets are all significantly associated with the KEGG pathway: Pathways in cancer. Since the three datasets are all cancer datasets, the results demonstrate that the predicted targets are closely related to the biological conditions of the three datasets. The detailed information of significant KEGG pathways in the three datasets can be found in S5 File.

**Table 3:** Top 10 enrichment KEGG pathways in the EMT, MCC and BR51 datasets. The *p*-values have been adjusted by Benjamini-Hochberg (BH) method.

**Datasets**	**Top 10 enrichment KEGG pathways**	**Adj-*p*-value**
EMT	Tight junction	3.43E-05
	Arrhythmogenic right ventricular cardiomyopathy	6.56E-05
	Leukocyte transendothelial migration	0.000602
	Cell adhesion molecules (CAMs)	0.000941
	ECM-receptor interaction	0.004284
	Pathways in cancer	0.006845
	Focal adhesion	0.009677
	Adherens junction	0.010311
	Endocytosis	0.010872
	Pathogenic Escherichia coli infection	0.020611
MCC	Vascular smooth muscle contraction	2.18E-05
	PPAR signaling pathway	2.62E-05
	Prostate cancer	7.71E-05
	Focal adhesion	0.00192
	Cytokine-cytokine receptor interaction	0.002144
	Oocyte meiosis	0.002232
	Alzheimer’s disease	0.002648
	Adherens junction	0.002803
	Mineral absorption	0.002821
	Pathways in cancer	0.003993
BR51	Cytokine-cytokine receptor interaction	1.49E-05
	Pathways in cancer	2.52E-05
	Chemokine signaling pathway	0.000217
	Focal adhesion	0.000234
	NOD-like receptor signaling pathway	0.000267
	Complement and coagulation cascades	0.00043
	Chagas disease (American trypanosomiasis)	0.000741
	Amoebiasis	0.000741
	Endocytosis	0.000741
	Leukocyte transendothelial migration	0.001382

### Highly-confident novel interactions

**Fig 5:**
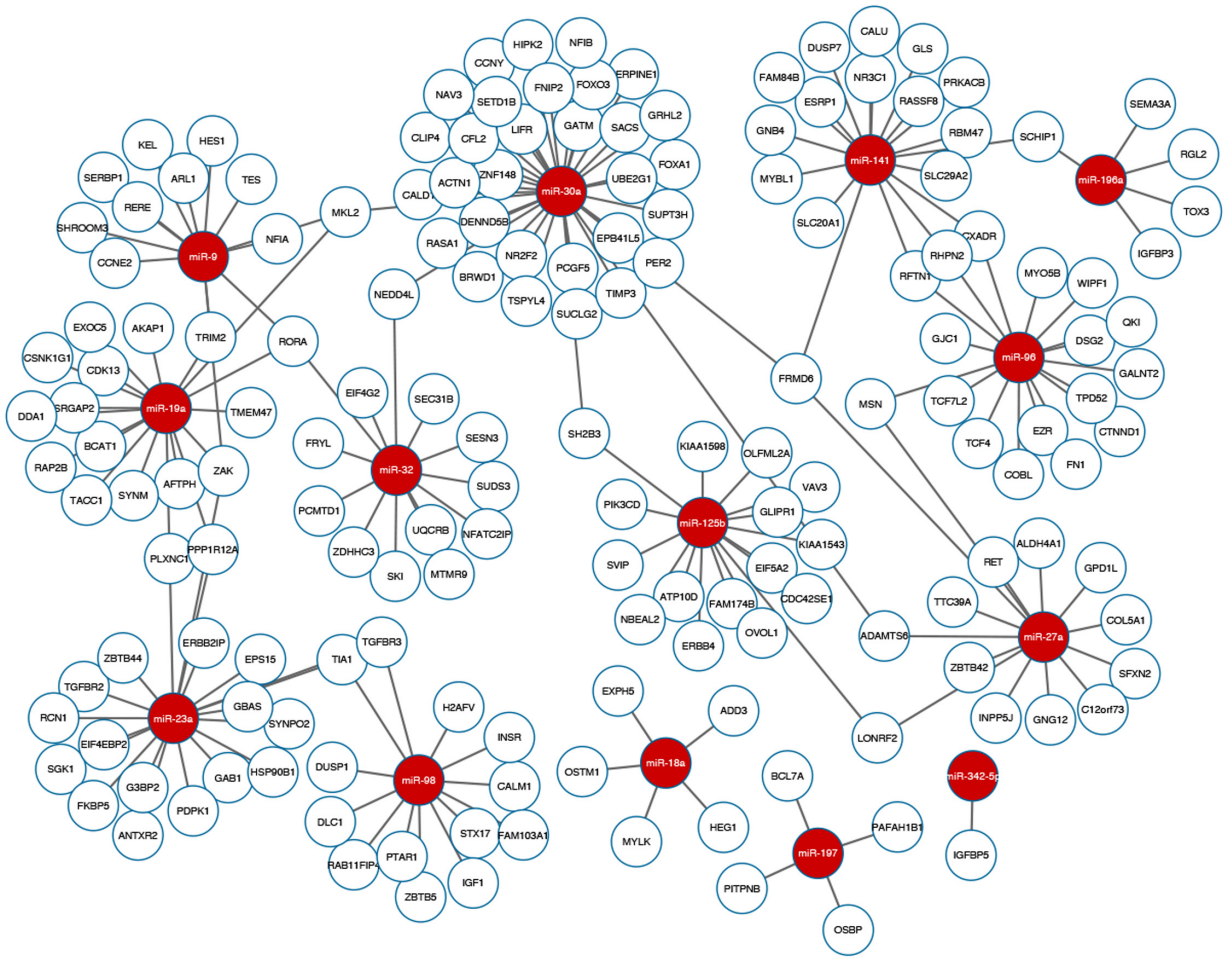
Highly-confident novel interactions in the EMT, MCC, and BR51 datasets. Red nodes are miRNAs and white nodes are mRNAs. The interactions are predicted by both the ensemble method, Pearson+IDA+Lasso, and TargetScan.

As experimentally confirmed databases are still sparse, we report a set of highly-confident novel interactions for further experiment validations. The novel interactions are those predicted by Pearson+IDA+Lasso and included in TargetScan v6.2 [61], but not yet confirmed by the experimentally confirmed databases. While the ensemble method provides reliable results based on expression data, TargetScan v6.2 provides predictions on direct binding information using sequence data. Integrating the effects measured by expression levels and the sequence binding information would provide highly-confident candidates for followed-up experiments. Fig 5 shows the novel interactions in the three datasets, where red nodes are miRNAs and white nodes are novel target genes. Details of the highly-confident novel interactions are in S6 File.

## Discussion and conclusion

The huge amount of high-throughput expression data calls for novel computational methods for predicting miRNA targets. Recently, a wide range of computational methods have been proposed to identify miRNA targets from expression data. However, each method has its advantages and limitations, and the performance of a method is not consistent across different datasets. Thus, how to obtain reliable and comprehensive results is a long-standing challenge for miRNA target prediction.

In this paper, we are inspired by the concept of *The wisdom of crowds* in [32] to propose ensemble methods. We firstly collect 8 popular miRNA target prediction methods (Pearson, IDA, MIC, Lasso, Elastic, Z-score, ProMISe and GenMiR++) to compare the performance of them using the real world gene expression datasets. We then develop ensemble methods using Borda rank election to combine the rankings from different methods. As shown in the Results section, the ensemble methods are better or as good as the individual methods. While the results reported in [32] showing the outperformance of the ensemble method over individual ones for predicting TF-gene interactions, here we also show similar results that can be achieved for the problem of miRNA target prediction.

Ensemble methods help with obtaining more comprehensive results than individual methods. However, combining more individual methods does not guarantee better performance of the ensemble method. For example, the level 3 ensemble method (Pearson+IDA+Lasso) performs the best in the EMT, MCC and BR51 datasets rather than the level 5 ensemble method (Pearson+IDA+MIC+Lasso+Z-score). The experimental results also suggest that combining the methods taking different approaches may result in a better ensemble method than combining methods in the same category, e.g. combining methods using correlation analysis. A possible reason is that the complementary behavior of the ensemble methods can be best utilised in those cases. The software tool we provided in this paper includes source codes of eight prediction methods, and the ensemble method whose procedure is described in the Methods section. The tool will help researchers apply the ensemble method to new datasets, e.g. using Pearson+IDA+Lasso, which is the best method suggested by the results of this paper. The tool also allows researchers to create new ensemble methods when the novel single computational methods arise.

In summary, we propose a framework based on Borda count election to generate ensemble methods from existing individual methods. The results show that the ensemble method Pearson+IDA+Lasso is a good method for miRNA target prediction. We report high-confident novel miRNA targets for further experimental validations. The *R* codes are provided, so that future methods can be integrated to form novel ensemble methods.

## Supporting Information

S1 File Differential expression profiles of miRNAs and mRNAs for the EMT, MCC and BR51 datasets. The *p*-values are adjusted by Benjamini-Hochberg (BH) method.
(XLSX)

S2 File R source codes for eight individual miRNA target prediction methods and three ensemble approaches. (ZIP)

S3 File The union of four databases including TarBase, miRecords, miRWalk and miRTarBase. The total number of miRNA-mRNA interactions is 62858.
(XLSX)

S4 File Comparison between ensemble methods and part of confirmed miRNA-mRNA interactions by the ensemble method (Pearson+IDA+Lasso) and three individual methods (Pearson, IDA and Lasso) in EMT, MCC and BR51 datasets. Pearson+IDA+Lasso finds complementary results.
(XLSX)

S5 File The significant KEGG pathways of the prediction results using the ensemble method (Pearson+IDA+Lasso) in the EMT, MCC and BR51 datasets. The *p*-values are adjusted by Benjamini-Hochberg (BH) method.
(XLSX)

S6 File Highly-confident novel miRNA-mRNA interactions in the EMT, MCC and BR51 datasets. (XLSX)
